# Clinical Usefulness of Bioavailable Vitamin D and Impact of *GC* Genotyping on the Determination of Bioavailable Vitamin D in a Korean Population

**DOI:** 10.1155/2019/9120467

**Published:** 2019-01-13

**Authors:** Hyun-Young Kim, Jin Hyun Kim, Myeong Hee Jung, In Ae Cho, Youngjin Kim, Min-Chul Cho

**Affiliations:** ^1^Department of Laboratory Medicine, Gyeongsang National University Hospital, Gyeongsang National University College of Medicine, Jinju 52727, Republic of Korea; ^2^Biomedical Research Institute, Gyeongsang National University Hospital, Jinju 52727, Republic of Korea; ^3^Institute of Health Science, Gyeongsang National University, Jinju 52727, Republic of Korea; ^4^Department of Obstetrics and Gynecology, Gyeongsang National University Hospital, Gyeongsang National University College of Medicine, Jinju 52727, Republic of Korea; ^5^Department of Laboratory Medicine, Kyung Hee University Hospital, Kyung Hee University School of Medicine, Seoul 02447, Republic of Korea

## Abstract

**Background:**

Bioavailable 25-hydroxy vitamin D (25(OH)D) has been suggested for the accurate determination of vitamin D status. The purpose of this study was to determine the utility of bioavailable 25(OH)D in assessing vitamin D status when vitamin D-binding protein (VDBP) was significantly altered by pregnancy and liver cirrhosis (LC). The role of genotyping of *GC*, a gene encoding VDBP, in the determination of bioavailable 25(OH)D concentration in a Korean population was also evaluated.

**Methods:**

This prospective study enrolled a total of 136 subjects (53 healthy controls, 45 patients with LC, and 38 pregnant women) from 2017 to 2018. The concentrations of total 25(OH)D and VDBP were measured, and bioavailable 25(OH)D concentrations were calculated. *GC* genotyping was performed to determine rs4588 and rs7041 polymorphisms. Clinical and laboratory data were compared among the three groups of subjects.

**Results:**

Median VDBP and total 25(OH)D concentrations were 165.2 *μ*g/ml and 18.5 ng/ml in healthy controls, 76.9 *μ*g/ml and 10.5 ng/ml in patients with LC, and 368.9 *μ*g/ml and 17.7 ng/ml in pregnant women, respectively. Compared with controls, patients diagnosed with LC had significantly lower VDBP and total 25(OH)D concentrations (all *P* < 0.001) while pregnant women had significantly higher VDBP concentrations (*P* < 0.001). Although total 25(OH)D concentrations in pregnant women were similar to those in controls (*P* = 0.394), their bioavailable 25(OH)D concentrations were significantly lower (1.2 vs. 3.0 ng/ml; *P* < 0.001). Among all the three groups combined, the genotype-specific bioavailable 25(OH)D and the genotype-independent bioavailable 25(OH)D concentrations did not differ significantly (*P* = 0.299).

**Conclusions:**

Our study has demonstrated that bioavailable 25(OH)D concentration reflects vitamin D status more accurately than the total 25(OH)D concentration, especially in pregnant women. In addition, *GC* genotyping did not significantly affect bioavailable 25(OH)D concentration. Therefore, if VDBP concentration is significantly altered, the measurement of bioavailable 25(OH)D concentration might facilitate the accurate determination of vitamin D status. However, *GC* genotyping might be unnecessary.

## 1. Introduction

Humans synthesize vitamin D in skin following exposure to sunlight. Vitamin D can also be obtained from diet. Vitamin D does not exhibit biological activity until a two-step hydroxylation occurs. Following hydroxylation in the liver to 25-hydroxy vitamin D (25(OH)D), the vitamin D metabolite is transported to kidneys where it is converted to 1*α*, 25-dihydroxyvitamin D (1*α*, 25(OH)_2_D) [1, 2]. Active forms of vitamin D exhibit varying function in tissues and organs throughout the body. Most (85%–90%) of the circulating vitamin D is tightly bound to vitamin D-binding protein (VDBP) and only a smaller amount (10%–15%) is loosely bound to albumin. Less than 1% of circulating vitamin D exists in free unbound form [3–5]. The fraction that is not bound to VDBP (free and albumin-bound) is considered bioavailable 25(OH)D [3].

Currently, vitamin D status is assessed via measurement of the total 25(OH)D concentrations. The commonly used criteria for vitamin D status are as follows: vitamin D deficiency (<20 ng/ml) and vitamin D insufficiency (20–30 ng/ml) [2, 6, 7]. However, a few studies have reported a stronger correlation between serum calcium, parathyroid hormone [3], bone mineral density [8], and vascular outcomes [9] with bioavailable 25(OH)D concentration compared with total 25(OH)D concentration, suggesting the clinical significance of bioavailable 25(OH)D concentration. However, other studies have failed to detect stronger associations between bioavailable 25(OH)D concentration and clinical outcomes [10]. Thus, the clinical utility of bioavailable 25(OH)D in the assessment of vitamin D status remains unclear.

The bioavailable 25(OH)D concentration is affected by serum VDBP concentration and *GC* genotype. The VDBP concentration is altered depending on various conditions. For example, VDBP is increased by up to 50% under elevated estrogen levels (e.g., pregnancy), whereas it is decreased in certain disease states (e.g., severe hepatic disease) [11–14]. The *GC* gene encoding VDBP exhibits more than 120 polymorphisms. Two single nucleotide polymorphisms (SNPs), rs7041 and rs4588, generate three major polymorphic isoforms of VDBP: *Gc1f*, *Gc1s*, and *Gc2* [15, 16]. Because the affinity of VDBP for 25(OH)D depends on the polymorphic isoform, the *GC* genotype may also play a significant role in determining bioavailable 25(OH)D [13, 14, 17]. However, *GC* genotyping entails DNA extraction and PCR that are expensive, labor-intensive, and time-consuming procedures. To date, no published studies have evaluated the necessity or the impact of *GC* genotyping on bioavailable 25(OH)D in a Korean population.

Thus, the objectives of this study were (1) to determine whether calculation of bioavailable vitamin D might facilitate the assessment of vitamin D status in individuals with altered VDBP concentrations such as pregnant women and patients with liver cirrhosis (LC) and (2) evaluate whether *GC* genotyping might be essential for the determination of bioavailable 25(OH)D in Koreans.

## 2. Materials and Methods

### 2.1. Study Subjects

This prospective study enrolled a total of 136 subjects from March 2017 to March 2018, including 53 healthy individuals who underwent general medical check-ups without any symptoms, 45 patients with LC, and 38 pregnant women. In case of pregnant women, those with twins or triplets were excluded. Clinical and laboratory data including age, sex, albumin concentration, gestational age, and/or Child-Pugh class [18] were collected from electronic medical records. At the time of study enrollment, blood samples were collected and serum and leukocytes were separated and stored at -80°C. The study protocol was approved by the Institutional Review Board (IRB) of Gyeongsang National University Hospital (approval number: 2017-01-005). Written informed consent was obtained from all participants.

### 2.2. VDBP and Total 25(OH)D Assays

VDBP concentration was measured using an enzyme-linked immunosorbent assay (ELISA) kit (R&D Systems, Minneapolis, MN, USA) according to the manufacturer's protocol. Coefficients of variation (CVs) for three concentrations (10.9, 31.7, and 63.7 *μ*g/ml) by intra- and interassay were 5.7­6.2% and 5.1­7.4%, respectively [19]. Total 25(OH)D concentration was measured using an Elecsys vitamin D total electrochemiluminescence binding assay (Roche Diagnostics, Mannheim, Germany) and a Cobas 8000 e602 analyzer (Roche Diagnostics). CVs for four concentrations (6.8, 15.0, 28.0, and 67.0 ng/ml) of intra- and interassay were 1.7-7.8% and 2.2­10.7%, respectively [20].

### 2.3. GC Genotyping

Genomic DNA was isolated from peripheral blood leukocytes using a DNeasy Blood and Tissue Kit (Qiagen, Hilden, Germany) according to the manufacturer's instructions. *GC* genotyping for rs7041 (NM_000583.3:c.1296T>G; NP_000574.2:p.Asp432Glu) and rs4588 (c.1307C>A; p.Thr436Lys) was performed using TaqMan SNP Genotyping Assay (Thermo Fisher Scientific, Waltham, MA, USA) and an ABI ViiA 7 Real-Time PCR System (Applied Biosystems, Foster City, CA, USA) according to the manufacturer's instructions. Common *GC* alleles were determined as follows: *Gc1f* (c.1296T; c.1307C), *Gc1s* (c.1296G; c.1307C), and *Gc2* (c.1296T; c.1307A).

### 2.4. Calculation of Bioavailable 25(OH)D

Based on total 25(OH)D, VDBP, and albumin concentrations, bioavailable 25(OH)D concentrations were calculated using the following equations [17]:

(1) $$\begin{array}{c} Bioavailable\;25\left(OH\right)D=albumin‐bound\;25\left(OH\right)D+free\;25\left(OH\right)D=\left(albumin\times K_{albumin}+1\right)\times free\;25\left(OH\right)D,\\ Free\;25\left(OH\right)D=\frac{-b+\sqrt{b^{2}-4ac}}{2a},\\ a=K_{VDBP}\times K_{albumin}\times albumin+K_{VDBP},\\ b=K_{VDBP}\times VDBP-K_{VDBP}\times total\;25\left(OH\right)D+K_{albumin}\times albumin+1,\\ c=-Total\;25\left(OH\right)D,\\ K_{albumin}=6\times 10^{5}M^{-1},\\ K_{VDBP}\left[\text{for }genotype‐independent\;bioavailable\;25\left(OH\right)D\right]=0.7\times 10^{9}M^{-1}. \end{array}$$

To calculate genotype-specific bioavailable 25(OH)D concentrations, the variable *K*_VDBP_ was replaced by genotype-specific VDBP binding affinity (*K*_VDBP1f_, 1.12 × 10^9^ M^−1^; *K*_VDBP1s_, 0.6 × 10^9^ M^−1^; and *K*_VDBP2_, 0.36 × 10^9^ M^−1^) [15]. For heterozygous genotypes, mean affinity for the two homozygotes was used (*K*_VDBP1f/1s_, 0.86 × 10^9^ M^−1^; *K*_VDBP1f/2_, 0.74 × 10^9^ M^−1^; and *K*_VDBP1s/2_, 0.48 × 10^9^ M^−1^) [21]. In this study, “bioavailable 25(OH)D” refers to both genotype-independent and genotype-specific bioavailable 25(OH)D.

### 2.5. Statistical Analysis

We compared clinical and laboratory data and *GC* genotypes between groups using the chi-square test for categorical variables and the Kruskal-Wallis test for continuous variables, with the Tukey test based on ranks as post hoc analysis. Patient characteristics (Child-Pugh class or trimester) were compared using the Mann–Whitney test or two-sample *t*-test. *GC* genotype and allele frequencies were compared between groups using the chi-square test or Fisher's exact test. Genotype-independent and genotype-specific bioavailable 25(OH)D concentrations were compared using the Wilcoxon signed-rank test. Percent difference between genotype-independent and genotype-specific bioavailable 25(OH)D was calculated as [genotype-specific bioavailable 25(OH)D − genotype-independent bioavailable 25(OH)D]/genotype-independent bioavailable 25(OH)D × 100. The relationship between continuous variables was evaluated by Spearman's correlation. Values were expressed as median and interquartile range (IQR). *P* < 0.05 was considered significant. All statistical analyses were performed using IBM SPSS Statistics version 21 (IBM Corp., Armonk, NY, USA).

## 3. Results

### 3.1. Subject Characteristics

Median age of subjects was 50 years (IQR, 40–60 years) for healthy controls, 52 years (IQR, 46–62 years) for patients with LC, and 33 years (IQR, 32–36 years) for pregnant women. The proportion of women was 35.8% in the control group and 17.8% in the LC group. Albumin concentration was significantly lower in patients with LC (2.9 g/dl) than that in healthy controls (4.5 g/dl) and pregnant women (3.6 g/dl; both *P* < 0.001). Of the 45 patients with LC, 4 patients were Child-Pugh class A, 26 patients were class B, and 15 patients were class C. Of the 38 pregnant women, 2 were in the first trimester, 4 were in the second trimester, and 32 were in the third trimester (Table 1).

### 3.2. VDBP, Total 25(OH)D, and Bioavailable 25(OH)D Concentrations according to Medical Status

**Figure 1:**
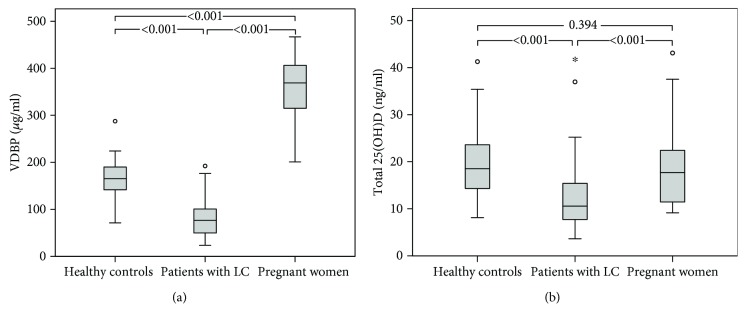
Box plots comparing (a) VDBP and (b) total 25(OH)D concentrations according to medical status. LC: liver cirrhosis; VDBP: vitamin D-binding protein; 25(OH)D: 25-hydroxy vitamin D.

**Figure 2:**
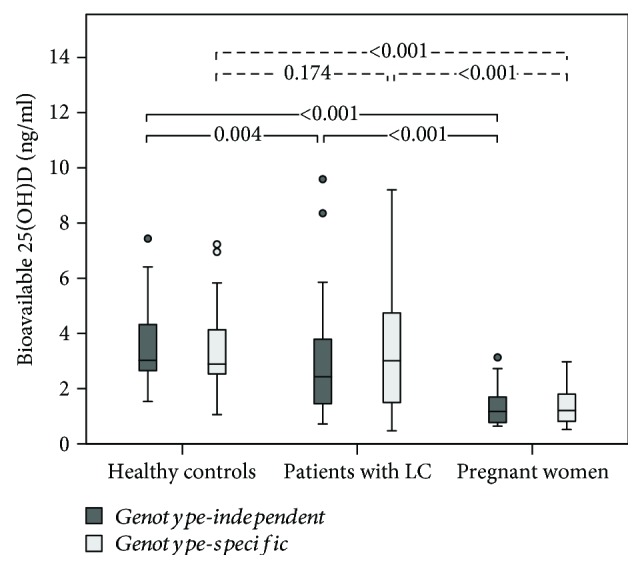
Box plots comparing genotype-independent and genotype-specific bioavailable 25(OH)D concentrations according to medical status. Genotype-independent and genotype-specific bioavailable 25(OH)D concentrations did not differ significantly for healthy controls, patients with LC, or pregnant women (all *P* > 0.05). LC: liver cirrhosis; VDBP: vitamin D-binding protein; 25(OH)D: 25-hydroxy vitamin D.

Circulating VDBP concentration was the highest in pregnant women (368.9 *μ*g/ml), followed by that in healthy controls (165.2 *μ*g/ml) and patients with LC (76.9 *μ*g/ml; all *P* < 0.001) (Table 2 and Figure 1(a)). Total 25(OH)D concentrations were similar between healthy controls and pregnant women (18.5 ng/ml (IQR, 14.2–23.9 ng/ml) vs. 17.7 (IQR, 11.4–22.7 ng/ml); *P* = 0.394). In contrast, total 25(OH)D concentrations were significantly lower in patients with LC (10.5 ng/ml (IQR, 7.1–15.4 ng/ml)) than those in other groups (*P* < 0.001) (Table 2 and Figure 1(b)). When genotype was not taken into account, bioavailable 25(OH)D concentration was 3.0 ng/ml in healthy controls, 2.4 ng/ml in patients with LC, and 1.2 ng/ml in pregnant women. Its concentrations were significantly lower in patients with LC and pregnant women compared to that in controls (*P* < 0.05) (Table 2 and Figure 2).

### 3.3. Genotype-Independent and Genotype-Specific Bioavailable 25(OH)D Concentrations

Genotype-independent bioavailable 25(OH)D concentration did not differ significantly from genotype-specific bioavailable 25(OH)D for the three groups combined (2.4 vs. 2.5 ng/ml; *P* = 0.299) or for healthy controls (3.0 vs. 2.9 ng/ml; *P* = 0.073), patients with LC (2.4 vs. 3.0 ng/ml; *P* = 0.077), or pregnant women (1.2 vs. 1.2 ng/ml; *P* = 0.058) (Table 3 and Figure 2). Mean percent difference between genotype-specific bioavailable 25(OH)D concentration and genotype-independent bioavailable 25(OH)D was -0.9%, 8.4%, and -0.5% in controls, patients with LC, and pregnant women, respectively. Although total 25(OH)D concentration did not correlate with VDBP concentration in any of these groups (all *P* > 0.05) (Supplementary Table 1), total 25(OH)D concentration was significantly correlated with bioavailable 25(OH)D concentration in all groups. Moreover, the correlation between total 25(OH)D and genotype-independent bioavailable 25(OH)D was stronger than the correlation between total 25(OH)D and genotype-specific bioavailable 25(OH)D for each group (controls, *r* = 0.841 vs. *r* = 0.548; patients with LC, *r* = 0.808 vs. *r* = 0.703; and pregnant women, *r* = 0.888 vs. *r* = 0.643) (Supplementary Table 1).

### 3.4. VDBP, Total 25(OH)D, and Bioavailable 25(OH)D Concentrations according to Child-Pugh Class (Patients with LC) and Trimester (Pregnant Women)

In patients with LC, VDBP concentrations were significantly lower in patients with Child-Pugh class C than those in patients with Child-Pugh class A and B (45.3 vs. 91.6 *μ*g/ml; *P* < 0.001). However, total and bioavailable 25(OH)D concentrations did not differ significantly between these subgroups (Table 4(a)). In pregnant women, VDBP, total 25(OH)D, and bioavailable 25(OH)D concentrations in women in the first or second trimester did not differ significantly from those in women in the third trimester (Table 4(b)).

### 3.5. GC Genotype and Allele Frequencies and Serum Concentrations of VDBP, Total 25(OH)D, and Bioavailable 25(OH)D according to GC Genotype

The most common genotype in the three groups combined was *Gc1f/Gc2* (36.0%), followed by *Gc1f/Gc1s* (18.4%), *Gc1s/Gc2* (16.9%), *Gc1f/Gc1f* (14.7%), *Gc2/Gc2* (8.8%), and *Gc1s/Gc1s* (5.1%) (Table 5). *Gc1f/Gc2* was the most common genotype in healthy controls (34.0%) and pregnant women (47.4%), whereas *Gc1s/Gc2* was the most common genotype in patients with LC (31.1%). In patients with LC, *Gc1f/Gc1s* was less common while *Gc1s/Gc2* was more common compared to controls. In the three groups combined, frequencies of *Gc1f*, *Gc1s*, and *Gc2* were 41.9%, 22.8%, and 35.3%, respectively. The allele *Gc1f* was the most common in controls (46.2%) and pregnant women (44.7%) while *Gc2* was the most common in patients with LC (41.1%). However, VDBP, total 25(OH)D, and genotype-independent bioavailable 25(OH)D concentrations did not differ significantly according to *GC* genotype in healthy controls (Supplementary Table 2).

## 4. Discussion

In our study, the highest levels of serum VDBP concentrations were recorded in pregnant women and the lowest values were found in patients with LC as expected. The total 25(OH)D and genotype-independent bioavailable 25(OH)D concentrations were lower in patients with LC compared with those in healthy controls (*P* < 0.001 for both). This finding is consistent with previous studies reporting a high prevalence of total 25(OH)D deficiency in patients with chronic liver disease [14, 22–25]. The result could be attributed to hepatic dysfunction (e.g., liver cirrhosis) and lifestyle of chronic patients. In other words, impaired 25-hydroxylation of vitamin D and hepatic VDBP synthesis, decreased outdoor activity, low dietary intake of vitamin D, and poor absorption lead to low total 25(OH)D concentrations [26–28]. Despite the low levels of total 25(OH)D concentrations in patients with chronic liver disease, free 25(OH)D concentrations have been reported to be normal or increased in such patients compared with controls [14, 22, 24, 25]. However, little is known about bioavailable 25(OH)D in patients with liver cirrhosis.

Although the total 25(OH)D concentrations in pregnant women were similar to those in healthy controls in our study, their bioavailable 25(OH)D concentrations were significantly lower than in controls. Previous studies evaluating free or bioavailable 25(OH)D concentrations in pregnant women have yielded discrepant results. For example, Schwartz et al. [14, 25] have found no significant difference in total or free 25(OH)D concentrations between pregnant women and healthy controls. However, Kim et al. [29] have reported that bioavailable 25(OH)D concentrations were significantly lower in pregnant women (1.9 vs. 2.6 ng/ml; *P* = 0.003), although the total 25(OH)D concentrations were comparable between pregnant women and healthy controls (18.3 vs. 18.3 ng/ml; *P* = 0.808), similar to our results. Most previous studies assessing bioavailable vitamin D in pregnant women have enrolled Caucasian and African-American participants. The vitamin D status of ethnic minorities has been less studied [30], and few studies have enrolled Korean participants. Thus, differences in ethnicity may account for these discrepant results, which may be attributed to the effect of genetic polymorphisms in vitamin D metabolism and transport [30]. Our results imply that bioavailable 25(OH)D might reflect vitamin D status more accurately than the total 25(OH)D, especially in pregnant women. Thus, measurement of the bioavailable 25(OH)D levels may be essential to accurately assess vitamin D status, at least in pregnant Korean women. Ethnicity and pregnancy-specific vitamin D status need to be further evaluated.

The use of *GC* genotype-specific VDBP binding affinity values to calculate the bioavailable 25(OH)D was expected to reveal the differences between genotype-independent and genotype-specific values. Arnaud and Constans [15] have reported different VDBP affinities for total 25(OH)D according to the *GC* genotype. The affinity of *Gc1f* was fourfold higher than that of *Gc2* and twofold higher than that of *Gc1s*. However, in our study, the genotype-independent and genotype-specific bioavailable 25(OH)D concentrations did not differ significantly. In patients with LC, genotype-independent bioavailable 25(OH)D concentrations appeared to be lower than genotype-specific 25(OH)D concentrations (2.4 vs. 3.0 ng/ml), although the difference was not significant (*P* = 0.077). Our results indicate that the *GC* genotype has a limited effect on the calculated value of bioavailable 25(OH)D. In addition, we found that the correlation between total and genotype-independent bioavailable 25(OH)D concentrations was higher than the correlation between total and genotype-specific bioavailable 25(OH)D concentrations.

The minimal impact of *GC* genotype on the calculated bioavailable 25(OH)D concentration might be explained by the distribution of *GC* alleles in subjects enrolled in this study. To calculate genotype-independent bioavailable 25(OH)D, we used the binding affinity constant of VDBP (*K*_VDBP_), which represents the average affinity constant of three isoforms. In our study, the frequency of *GC* alleles in all subjects was 41.9% for *Gc1f*, 35.3% for *Gc2*, and 22.8% for *Gc1s*, which showed relatively even distribution, which may explain the lack of significant difference between genotype-independent bioavailable 25(OH)D calculated using the average *K*_VDBP_ and genotype-specific bioavailable 25(OH)D calculated using genotype-specific *K*_VDBP_. In other words, the even distribution of *GC* allele in the present study might arithmetically compensate the effect of genotype-specific *K*_VDBP_ on the calculation of bioavailable 25(OH)D. In fact, a previous study [31] of 360 Korean subjects has reported that the allele frequencies of *Gc1f*, *Gc1s*, and *Gc2* were 44%, 25%, and 31%, respectively, which were similar to our results. Thus, although our study included a relatively small number of subjects, our results suggesting a limited effect of the *GC* genotype on bioavailable 25(OH)D value may still be meaningful. However, further studies enrolling larger numbers of Korean subjects are needed to corroborate these results.

In our study, patients with severe hepatic dysfunction (Child-Pugh class C) had lower VDBP concentrations than patients with Child-Pugh class A and B (45.3 vs. 91.6 *μ*g/ml; *P* < 0.001). The total 25(OH)D appeared to be lower in patients with Child-Pugh class C, but not significant (8.6 vs. 11.7 ng/ml; *P* = 0.057). Bioavailable 25(OH)D concentrations did not differ between groups (all *P* > 0.05), indicating similar total and bioavailable 25(OH)D concentrations despite differences in hepatic dysfunction. Arteh et al. [23] have shown that severe total 25(OH)D deficiency is more common in patients diagnosed with LC than in patients with noncirrhotic liver disease. Lai et al. [24] have reported that in patients with LC, those with low albumin concentrations also carry lower concentrations of VDBP, total 25(OH)D, and free 25(OH)D than patients with normal albumin concentrations.

The analysis of VDBP, total 25(OH)D, and bioavailable 25(OH)D concentrations in pregnant women showed no significant differences according to the trimester. However, only two women in the first trimester were enrolled in our study. Therefore, we only compared the two groups (first and second trimesters vs. third trimester). Ma et al. demonstrated that VDBP, 1*α*-hydroxylase, 24-hydroxylase, and vitamin D receptor were expressed on the placenta [32], and Cleal et al. showed that maternal 25(OH)D and VDBP concentrations may mediate the regulation of amino acid transfer to the fetus [33], suggesting that dysregulation of VDBP as well as vitamin D could be a risk factor in the pregnancy outcome (i.e., preeclampsia, preterm birth, and gestational diabetes) [34]. Further studies are needed to analyze the VDBP, total 25(OH)D, and bioavailable 25(OH)D concentrations in pregnant women according to the trimester and pregnancy outcome.

The major *GC* genotype and allele frequencies are known to vary among ethnicities [16]. For example, Nielson et al. [35] have reported that nearly all African-American subjects and all Gambian subjects carry the *Gc1f* allele (*Gc1f/Gc1f*, *Gc1f/Gc1s*, or *Gc1f/Gc2*). In contrast, most white subjects did not harbor the *Gc1f* allele while *Gc1s/Gc1s* and *Gc1s/Gc2* were the most frequent genotypes in this group. Koreans carry different *GC* allele frequencies than African-Americans or whites. Jung et al. [31] have enrolled 203 patients with chronic obstructive pulmonary disease and 157 control subjects and reported that *Gc1f/Gc2* (25%) was the most frequent genotype, followed by *Gc1f/Gc1f* (22%), *Gc1f/Gc1s* (20%), and *Gc1s/Gc2* (18%), similar to our results. Furthermore, we observed different *GC* genotype frequencies in patients with LC compared with healthy controls. The *Gc1s/Gc2* genotype was more common in patients with LC than in controls (31.1% vs. 9.4%; *P* = 0.007), whereas *Gc1f/Gc1s* was less common (8.9% vs. 24.5%; *P* = 0.042).

Our study has a few limitations. First, we measured VDBP concentrations using monoclonal ELISA. Nielson et al. [35] have reported that VDBP concentrations measured with monoclonal ELISA are strongly correlated with *GC* genotypes. Therefore, this assay underestimated VDBP concentrations in African-Americans because of the high frequency of *Gc1f*. A study evaluating VDBP concentrations in Korean subjects by polyclonal ELISA may be necessary. Second, we have not used liquid chromatography-tandem mass spectrometry (LC-MS/MS) to measure the total 25(OH)D concentrations. However, in healthy controls, the measured total 25(OH)D concentration (18.5 ng/ml) was similar to that reported by a previous study (18.3 ng/ml) in Korea using LC-MS/MS [29]. Third, the small sample size limited our ability to determine the effect of *GC* genotype on VDBP concentrations and compare subgroups according to the Child-Pugh class in patients diagnosed with LC and trimester in pregnant women. Fourth, we have not investigated vitamin D supplementation or sun exposure. We have not considered seasonal variation either. Fifth, we failed to exclude subjects with other conditions (e.g., malnutrition, infection, and nephrotic syndrome) that could affect VDBP concentrations other than LC and pregnancy.

## 5. Conclusions

In this study, we demonstrated that bioavailable 25(OH)D levels reflect vitamin D status more accurately than the total 25(OH)D concentrations, especially in pregnant women. In addition, we have shown that the *GC* genotype did not significantly affect bioavailable 25(OH)D concentration in Koreans. Therefore, if the VDBP concentration is significantly altered, calculation of bioavailable 25(OH)D levels might facilitate the accurate determination of vitamin D status obviating the need for GC genotyping.

## Figures and Tables

**Table 1:** Subject characteristics.

	Healthy controls (*N* = 53)	Patients with LC (*N* = 45)	Pregnant women (*N* = 38)	*P* value
Age (yr)	50 (40-60)	52 (46-62)	33 (32-36)	<0.001
Female (*N*)	19 (35.8%)	8 (17.8%)	38 (100%)	<0.001
Albumin (g/dl)	4.5 (4.4-4.7)	2.9 (2.2-3.3)	3.6 (3.5-3.8)	<0.001
Child-Pugh class (*N*)				
A		4 (8.9%)		
B		26 (57.8%)		
C		15 (33.3%)		
Trimester (*N*)				
First			2 (5.3%)	
Second			4 (10.5%)	
Third			32 (84.2%)	

Values are presented with median (interquartile range) or number (percentage). yr: year; LC: liver cirrhosis.

**Table 2:** VDBP, total 25(OH)D, and genotype-independent bioavailable 25(OH)D concentrations according to medical status.

	Healthy controls (*N* = 53)	Patients with LC (*N* = 45)	Pregnant women (*N* = 38)	*P* value
VDBP (*μ*g/ml)	165.2 (140.5-191.6)	76.9 (49.8-101.4)	368.9 (313.8-407.6)	<0.001
Total 25(OH)D (ng/ml)	18.5 (14.2-23.9)	10.5 (7.1-15.4)	17.7 (11.4-22.7)	<0.001
Genotype-independent bioavailable 25(OH)D (ng/ml)	3.0 (2.6-4.3)	2.4 (1.4-3.9)	1.2 (0.8-1.7)	<0.001

Values are presented with median (interquartile range). LC: liver cirrhosis; VDBP: vitamin D-binding protein; 25(OH)D: 25-hydroxy vitamin D.

**Table 3:** Comparison of genotype-independent and genotype-specific bioavailable 25(OH)D concentrations according to medical status.

	Genotype-independent bioavailable 25(OH)D (ng/ml)	Genotype-specific bioavailable 25(OH)D (ng/ml)	Mean percent difference between genotype-independent and genotype-specific bioavailable 25(OH)D	*P* value
Total subjects (*N* = 136)	2.4 (1.4-3.6)	2.5 (1.3-3.8)	2.3%	0.299
Healthy controls (*N* = 53)	3.0 (2.6-4.3)	2.9 (2.5-4.2)	-0.9%	0.073
Patients with LC (*N* = 45)	2.4 (1.4-3.9)	3.0 (1.5-4.8)	8.4%	0.077
Pregnant women (*N* = 38)	1.2 (0.8-1.7)	1.2 (0.8-1.8)	-0.5%	0.058

Values are presented with median (interquartile range). LC: liver cirrhosis; 25(OH)D: 25-hydroxy vitamin D.

**Table:** (a) Patients with LC

	Child-Pugh class A and B (*N* = 30)	Child-Pugh class C (*N* = 15)	*P* value
VDBP (*μ*g/ml)	91.6 (73.0-119.8)	45.3 (33.4-52.6)	<0.001
Total 25(OH)D (ng/ml)	11.7 (8.2-17.7)	8.6 (5.9-12.6)	0.057
Bioavailable 25(OH)D (ng/ml)			
Genotype-independent	2.4 (1.4-4.0)	2.4 (1.2-3.6)	0.866
Genotype-specific	2.9 (1.2-4.4)	3.0 (1.5-4.9)	0.413

**Table:** (b) Pregnant women

	First and second trimester (*N* = 6)	Third trimester (*N* = 32)	*P* value
VDBP (*μ*g/ml)	341.1 (260.8-386.5)	371.1 (320.2-412.4)	0.213
Total 25(OH)D (ng/ml)	18.2 (12.9-23.5)	16.7 (11.3-23.2)	0.653
Bioavailable 25(OH)D (ng/ml)			
Genotype-independent	1.4 (1.1-2.1)	1.1 (0.8-1.7)	0.245
Genotype-specific	1.4 (1.0-2.0)	1.0 (0.8-1.7)	0.469

Values are presented with median (interquartile range). VDBP: vitamin D-binding protein; 25(OH)D: 25-hydroxy vitamin D; LC: liver cirrhosis.

**Table 5:** Major *GC* genotype and allele frequencies.

	Healthy controls (*N* = 53)	Patients with LC (*N* = 45)	*P* value^a^	Pregnant women (*N* = 38)	*P* value^b^	Total (*N* = 136)
*Genotype frequencies*						
*Gc1f/Gc1f*	9 (17.0%)	7 (15.6%)	0.849	4 (10.5%)	0.386	20 (14.7%)
*Gc1f/Gc1s*	13 (24.5%)	4 (8.9%)	0.042	8 (21.1%)	0.698	25 (18.4%)
*Gc1f/Gc2*	18 (34.0%)	13 (28.9%)	0.590	18 (47.4%)	0.197	49 (36.0%)
*Gc1s/Gc1s*	3 (5.7%)	2 (4.4%)	1.000	2 (5.3%)	1.000	7 (5.1%)
*Gc1s/Gc2*	5 (9.4%)	14 (31.1%)	0.007	4 (10.5%)	1.000	23 (16.9%)
*Gc2/Gc2*	5 (9.4%)	5 (11.1%)	1.000	2 (5.3%)	0.695	12 (8.8%)
*Allele frequencies*						
*Gc1f*	49 (46.2%)	31 (34.4%)	0.094	34 (44.7%)	0.842	114 (41.9%)
*Gc1s*	24 (22.6%)	22 (24.4%)	0.767	16 (21.1%)	0.799	62 (22.8%)
*Gc2*	33 (31.1%)	37 (41.1%)	0.146	26 (34.2%)	0.662	96 (35.3%)

In ^a^patients with LC and ^b^pregnant women, genotype and allele frequencies were compared with the healthy controls. LC: liver cirrhosis.

## Data Availability

The data used to support the findings of this study are available from the corresponding author upon request.

## Supplementary Materials

Supplementary Materials The supplementary word file contains two supplementary tables described in the text to support our results. Supplementary Table 1: Spearman's correlation coefficient between total 25(OH)D, VDBP, and bioavailable 25(OH)D. Supplementary Table 2: VDBP, total 25(OH)D, and bioavailable 25(OH)D concentrations according to GC genotype in healthy controls.
