# Supplementary material for: Clinical Usefulness of Bioavailable Vitamin D and Impact of GC Genotyping on the Determination of Bioavailable Vitamin D in a Korean Population
Source: Int J Endocrinol. 2019 Jan 13;2019:9120467. doi: 10.1155/2019/9120467 (PMC6350553; doi:10.1155/2019/9120467)
Supplement: Supplementary Materials — The supplementary word file contains two supplementary tables described in the text to support our results. Supplementary Table 1: Spearman's correlation coefficient between total 25(OH)D, VDBP, and bioavailable 25(OH)D. Supplementary Table 2: VDBP, total 25(OH)D, and bioavailable 25(OH)D concentrations according to GC genotype in healthy controls. [file 9120467.f1.docx]

**Supplementary Table 1.** Spearman correlation coefficient between total 25(OH)D, VDBP and bioavailable 25(OH)D

|  | Total 25(OH)D | Genotype-independent bioavailable 25(OH)D | Genotype-specific bioavailable 25(OH)D |
| --- | --- | --- | --- |
| **Total** |  |  |  |
| VDBP, μg/ml | 0.362 | -0.455 | -0.522 |
| Bioavailable 25(OH)D, ng/ml |  |  |  |
| Genotype-independent | 0.583 |  |  |
| Genotype-specific | 0.432 | 0.924 |  |
| **Healthy controls** |  |  |  |
| VDBP, μg/ml | 0.240 (n.s.) | -0.254 (n.s.) | -0.413 |
| Bioavailable 25(OH)D, ng/ml |  |  |  |
| Genotype-independent | 0.841 |  |  |
| Genotype-specific | 0.548 | 0.777 |  |
| **Patients with LC** |  |  |  |
| VDBP, μg/ml | 0.236 (n.s.) | -0.266 (n.s.) | -0.359 |
| Bioavailable 25(OH)D, ng/ml |  |  |  |
| Genotype-independent | 0.808 |  |  |
| Genotype-specific | 0.703 | 0.930 |  |
| **Pregnant women** |  |  |  |
| VDBP, μg/ml | 0.034 (n.s.) | -0.342 | -0.436 |
| Bioavailable 25(OH)D, ng/ml |  |  |  |
| Genotype-independent | 0.888 |  |  |
| Genotype-specific | 0.643 | 0.810 |  |

Abbreviations: VDBP, vitamin-D binding protein; 25(OH)D, 25-hydroxy vitamin D; LC, liver cirrhosis; n.s., no significance

**Supplementary Table 2.** VDBP, total 25(OH)D, and bioavailable 25(OH)D concentrations according to *GC* genotype in healthy controls

|  | VDBP, μg/ml | *P* value | Total 25(OH)D, ng/mL | *P* value | Genotype-independent bioavailable 25(OH)D, ng/ml | *P* value | Genotype-specific bioavailable 25(OH)D, ng/ml | *P* value |
| --- | --- | --- | --- | --- | --- | --- | --- | --- |
| *Gc1f/Gc1f (*N=9) | 174.1 (155.8-197.5) | 0.421 | 25.0 (16.2-27.6) | 0.267 | 3.7 (2.2-4.6) | 0.780 | 2.4 (1.5-3.1) | 0.035 |
| *Gc1f/Gc1s* (N=13) | 189.4 (144.0-208.4) |  | 20.9 (18.1-25.9) |  | 3.0 (2.5-4.9) |  | 2.5 (2.1-4.1) |  |
| *Gc1f/Gc2* (N=18) | 150.7 (136.8-180.7) |  | 17.4 (14.0-21.9) |  | 3.0 (2.7-4.1) |  | 2.9 (2.6-3.9) |  |
| *Gc1s/Gc1s* (N=3) | 162.7 (134.5-NA) |  | 13.3 (11.7-NA) |  | 2.3 (2.2-NA) |  | 2.6 (2.5-NA) |  |
| *Gc1s/Gc2* (N=5) | 154.4 (138.5-192.9) |  | 18.7 (16.1-20.1) |  | 3.2 (2.5-4.2) |  | 4.4 (3.4-5.5) |  |
| *Gc2/Gc2* (N=5) | 147.8 (139.2-173.3) |  | 14.9 (10.4-23.4) |  | 2.9 (1.8-4.3) |  | 4.8 (3.0-7.1) |  |

Values are presented with median (interquartile range).

Abbreviations: VDBP, vitamin-D binding protein; 25(OH)D, 25-hydroxy vitamin D
